# Transcriptome-wide identification of the WUSCHEL-related homeobox transcription factors and their expression patterns underlying drought and salt stress in *Populus euphratica*

**DOI:** 10.3389/fpls.2025.1747710

**Published:** 2026-01-30

**Authors:** Boniface Ndayambaza, Jianhua Si, Xin Zhao, Yingxue Zhao, Dongmeng Zhou, Bing Jia, Zijin Liu, Xue Bai, Boyang Wang

**Affiliations:** 1 Key Laboratory of Ecohydrology of Inland River Basin, Northwest Institute of Eco-Environment and Resources, Chinese Academy of Sciences, Lanzhou, China; 2 University of Chinese Academy of Sciences, Beijing, China; 3 Institutional Center for Shared Technologies and Facilities of NIEER, Chinese Academy of Sciences, Lanzhou, China

**Keywords:** expression pattern, drought stress, *Populus euphratica*, salt stress, transcriptome-wide, WOX transcription factor family genes

## Abstract

Drought and salinity are major abiotic stresses that severely affect plant growth and the productivity of woody plants. The WUSCHEL-related homeobox (WOX) transcription factors are central to the regulation of plant development and plant stress responses, yet their roles in the stress-resilient desert tree *Populus euphratica* remain unexplored. Here we performed a genome-wide study to identify and characterize the WOX gene family of *P. euphratica* species. A total of 18 *PeuWOX* genes were discovered and characterized phylogenetically as modern, intermediate, and ancient clades. Members within each clade exhibited strong conservation in gene structure and protein motifs. Promoter analysis revealed a significant enrichment of stress- and hormone-responsive cis-elements. The 10 chosen *PeuWOX* genes were found to be differentially regulated by drought and salt stress in a tissue-specific manner by expression patterns through qRT-PCR. In leaves, *PeuWOX1*, *PeuWOX2*, *PeuWOX4*, and *PeuWOX5* genes were sharply downregulated under salt stress, while *PeuWOX9*, *PeuWOX10*, and *PeuWOX17* genes were significantly upregulated during prolonged drought or salt exposure. In roots and stems, *PeuWOX5* and *PeuWOX10* genes were induced rapidly, which may lead to a process of adaptation such as adventitious rooting, but *PeuWOX4* and *PeuWOX17* were induced over the long term associated with the development of the vascular system and long-term acclimation. Our results provided evidence that the *PeuWOX* family participates in a refined regulatory network, which fine-tunes stem, root, and leaf development with response to abiotic stress. This study provides an important background to the molecular mechanisms of the exceptional resistance to stress across *P. euphratica* and provides candidate genes to improve the stress tolerance of woody plants.

## 1 Introduction

Abiotic stresses such as drought and soil salinity are among the most prominent environmental factors severely restricting agricultural and forestry productivity worldwide (Zhu, 2016). These stresses are further aggravated by climate change, deteriorating the land and presenting difficult conditions for plant growth and development, especially in arid and semi-arid drylands (Reed and Stringer, 2016). Currently, soil salinity has seriously impacted plant growth on over 6% (approximately 800 million hectares) of the world’s land (Li et al., 2025). Moreover, drought impairs the essential plant physiological mechanisms, including metabolism, photosynthesis, and cellular integrity, which is a major cause of ultimately stunted growth, shoot and root biomass reduction, and/or plant death (Farooq et al., 2009). To survive, plants may develop intricate systems to sense and respond to these harsh environments. An important trait to be targeted for improvement is reprogramming of the root system architecture such that it enhances increased water and nutrient acquisition under stress condition (Mohammadi Alagoz et al., 2023). These morphological responses, such as root resizing, rebranching, and hair development, play a key role in dealing with a water-limited environment (Rahman et al., 2021). Some plants, like trees, including *Populus*, have developed characteristics that enable them to be greatly tolerant to drought and salinity in soil. The desert poplar (*Populus euphratica*) is a unique tree that survives harsh desert conditions through remarkable stress tolerance (Miao et al., 2020). This typical poplar species is distributed in arid and saline-alkali regions of Northwest of China, especially Inner Mongolia, and exhibits strong resistance against high soil salinity and severe drought (Li et al., 2023; Zhang et al., 2023b). As such, it has been established as an excellent model to study the genetic and molecular mechanisms underlying abiotic stress response and adaptation in woody trees (Chen et al., 2015; Ma et al., 2013). Research on this species contributes directly to our understanding and addresses the issue of combating desertification and promoting ecosystem restoration (Mamat et al., 2018). Understanding the genetic regulation of these developmental traits is thus critical to breed stress-tolerant crops and tree species.

The WUSCHEL-related homeobox (WOX) transcription factor represents a plant-specific master regulator of stem cell maintenance and cell fate specification, which has crucial functions during plant growth and development (Dolzblasz et al., 2016; Fambrini et al., 2022). It includes the roles of embryogenesis, organ differentiation, and stress response onto crops and trees species (Wu et al., 2019). WOX proteins share a conserved homeodomain (HD) that facilitates binding to the specific DNA sequences of target genes and control their expression (van der Graaff et al., 2009; Zhou et al., 2023). The WOX proteins contain supplemental residues between helix 1 and helix 2 and between helix 2 and helix 3, whereas the WUS-box motif comprises eight conserved residues in the C-terminal of the HD. During the phylogenetic process of the higher plants, given the conserved domain and similarity of full-length amino acid sequence, 15 members of the WOX family in *Arabidopsis thaliana* genomes can be categorized into three clades, namely: (a) WUS clade, (b) intermediate clade, and (c) ancient clade. In *Arabidopsis thaliana*, the members of WOX gene family play a vital role in maintaining root apical meristem (RAM), shoot apical meristem (SAM), and vascular development, thus maintaining the balance between stem cell proliferation and differentiation (Haecker et al., 2004; Sarkar et al., 2007)—for example, *AtWOX4* activates vascular cambium activity and secondary growth in the pathway of CLE41 (Forzani et al., 2014; Hirakawa et al., 2010), whereas functionally redundant *AtWOX11/12* are essential for *de novo* adventitious root formation (Liu et al., 2014b). The archetypal function of WOX genes in meristem activity and organogenesis is conserved in spermatophytes, including trees like *Populus* (Kucukoglu et al., 2017). This process is unique due to the combination of primary organogenesis with secondary growth (Rasheed et al., 2024). The formation and maintenance of the RAM and SAM as well as all aerial organs are regulated by the genes of the recent WUS clade—for instance, in poplar (*Populus* ssp.), *PtoWUSa* is not only expressed in the organizing center to modulate the stem cell niche for indeterminate growth but also expressed in adventitious roots (Li et al., 2020). In addition, the shoot regeneration and differentiation of *Populus euphratica* Oliv. was investigated, which confirms an ideal molecular mechanism potentially controlling shoot differentiation in this species (Fu et al., 2019).

Previous genome-wide studies have expanded our understanding of the WOX gene transcription factor family in various tree species, underlining their conserved significance in plant growth and stress adaptation. Interestingly, WOX11 and WOX12 are the key regulators of drought and salt tolerance in trees—for instance, *PagWOX11/12a* enhances drought tolerance in poplar by promoting root growth. Its expression is activated by the upstream regulator *PagERF35* (Liu et al., 2021; Zhou et al., 2024). These significant genes enhance stress tolerance primarily by promoting root development, such as increasing root biomass, elongation, and root hair formation, which improves water and nutrient uptake (Rogers and Benfey, 2015). In addition, *MdWOX13–1* suggested drought tolerance by repressing the direct binding to the *MdMnSOD* promoter, and an increase of the callus weight and enhanced ROS scavenging were observed against drought stress (Lv et al., 2023). On the other hand, *PdbWOX4* negatively regulated the salt tolerance of the Shanxin poplar by repressing *PdbDREB2C*, which revealed that PdbWOX4 has a significant role in Shanxin poplar under salt stress (Li et al., 2025). Previous studies of WOX transcription factor members have been employed and reported in different plants, including Chinese native poplar (*Populus tumentosa*) (Liu et al., 2014a), paper mulberry (*Broussonetia kazinoki* × *Broussonetia papyrifera*) (Tang et al., 2017), walnuts (*Juglans regia* L.) (Chang et al., 2019), riparian shrub willow (*Salix suchowensis*) (Wang et al., 2019), black cottonwood (*Populus trichocarpa*) (Wang Shuang et al., 2019), citrus (*Citrus sinensis*) (Shafique Khan et al., 2021), longan (*Dimocarpus longan*) (Ma et al., 2022), apple (*Malus domestica*) (Lv et al., 2023), eucalyptus (*Eucalyptus grandis*) (Chen et al., 2024), Masson pine (*Pinus massoniana*) (Wang et al., 2024), Asian pear (*Pyrus pyrifolia*) (Liu et al., 2025), Chinese gugertree (*Schima superba*) (Jia et al., 2025), Korean pine (*Pinus koraiensis*) (Zhang et al., 2025), Shanxin poplar (*Populus davidiana* × *P. bolleana*) (Li et al., 2025), etc. However, a comprehensive profile of WOX genes remains unclear in many other Salicaceae species and their involvement in drought and salt stress responses, such as desert poplar (*Populus euphratica*) (Rasheed et al., 2024). A deeper understanding of these genes is therefore needed to explore a theoretical basis for boosting abiotic stress adaptability and shed light on ecosystem restoration in arid areas.

In this research, we performed a genome-wide identification and systematic analysis of the WOX transcription factor family in *P. euphratica*. A total of 18 *PeuWOX* genes were identified and systematically investigated in terms of their phylogenetic relationships, gene structures, conserved protein motifs, chromosome localization, and promoter cis-elements. In addition, we examined their expression patterns in different tissues and under drought and salt treatments by qRT-PCR analysis. The results serve as key groundwork for further dissecting the functional analysis of the *PeuWOX* transcription factor family, especially in response to abiotic stress hormones driving root development and growth adaptation, and they also accelerated genetic improvement using candidate genes to enhance resistance against stresses in poplar and closely related woody plants.

## 2 Materials and methods

### 2.1 Data retrial and identification of WOX genes in *P. euphratica* species

Genomic sequences of *P. euphratica* were downloaded from the NCBI databank (https://www.ncbi.nlm.nih.gov/protein, accessed on 28 July 2025) by keyword search with the keywords “WOX and *Populus euphratica*”. The protein sequences of *A. thaliana* were obtained from (Xu et al., 2025), and *P. trichocarpa* genome data were according to published reports (Hou et al., 2021). To identify the WOX gene members of *P. euphratica*, deduced amino acid sequences of 15 AtWOXs were used as queries to search all of the protein sequences of *P. euphratica* using BLASTP software (https://blast.ncbi.nlm.nih.gov/Bast.cgi; accessed on July 28, 2025). For the verification of all identified WOX members, we applied the pfam protein family database (Mistry et al., 2021) to eliminate redundancies based on Hidden Motion Model (HMM) method under the WOX structural domain (PF00046) (accessed July 28, 2025). The online expasy tools protParam (https://web.expasy.org/protparam/, accessed on July 29, 2025) were used to predict the physio-chemical properties including molecular weight (MW), instability index, iso-electric point (pI) at neutral pH, and GRAVY index for all of the new WOX family members identified in *P. euphratica*.

### 2.2 Phylogenetic tree construction, conserved motifs, gene structure, and cis-acting elements in the promoter analysis

To gain further understanding on the classification of the PeuWOX protein putatives, multiple sequence alignments were performed by using MEGA11 (default parameters), and a phylogenetic tree was constructed based on neighbor-joining method with bootstrap of 1,000 (Tamura et al., 2021). MEGA11 with the ClustalW function was also used to re-evaluate the characteristic structural domain of PeuWOX protein sequences and adjust the amino acid sequence. The phylogenetic tree was constructed by using the iTOL tool (v6) (Letunic and Bork, 2024). To visualize the intron–exon structure of PeuWOXs, MEME motif tool finder (v 5.5.7) was projected with default parameters (Bailey et al., 2009), and the maximum number of motifs was set to 10. Then, the gene structure and exon–intron structure of the PeuWOX proteins was identified using TBtools-II (Chen et al., 2023). In addition, the putative cis-acting regulatory elements for the promoter sequences were analyzed by submitting 18 *PeuWOX* genes to the PlantCARE database (Lescot et al., 2002).

### 2.3 Chromosomal location and synteny analysis

TBtools-II was used to locate the chromosomal positions of *PeuWOX* genes. The MCScanX software was used to analyze the gene duplication events (default parameters were used). Additionally, a syntenic relationship of WOX genes in *P. euphratica* with those of four other species (*P. deltoides*, *P. tremula* × *P. alba*, and *P. trichocarpa*) was constructed in this study using the Dual Synteny Plotter tool of TBtools-II.

### 2.4 Plant material treatments, RNA extraction, and qRT-PCR analysis

Two-year-old *P. euphratica* seedlings were potted under controlled conditions as established by Han et al. (2021). A total of 12 consistent adopted seedlings were then taken, with four for each treatment. For salt stress treatment, the seedlings were watered with 200 mM of NaCl solution for 2 weeks (Rajput et al., 2015), while the control seedlings were maintained with regular watering. Following exposure to stress for durations of 0, 24, 48, and 96 h, root, stem, and leaf tissues were sampled from a minimum of three seedlings per time point for subsequent RNA isolation. For drought stress, uniformly grown 2-year-old *P. euphratica* seedlings were harvested in Inner Mongolia and in Alxa region in China and placed in plastic pots (36 cm round on top and 40 cm deep) with a packet of potting soil, peat moss, and sand (2:2:1, v/v) and allowed to grow for a period of 4 months under controlled environmental conditions. In the nondestructive monitoring of the physiological parameters of the entire experiment, a batch of 15 plants consisted of five controls and 10 water-deprived plants (separated into three groups, each group comprised of five individual biological replicates) was used in this study. Watering was done to field capacity daily (with group 0 as control). The treatment of water stress was introduced to model the slow process of soil drying that would mimic the occurrence of a natural drought. On the first day, watering was stopped in group 1, and this (without watering) continued for 10 days. Group 2 was kept without irrigating the soil in 5 days. The volumetric water content (VWC) of the soil was determined by using an oven (by drying soil samples at 105 °C). Samples of leaf, root, and stem tissues were collected for the control and 5- and 10-day treatments, respectively, according to the procedures of Tang et al. (2013). Each experimental treatment was replicated three times independently. All samples collected (0.2 g) were frozen in liquid nitrogen and kept at −80 °C until use. Total RNA from desert poplar roots, stems, and leaves was extracted using RNeasy Mini Kit (Qiagen, Hilden, Germany). RNA quality was tested by gel electrophoresis, and purity (A260/A280 = 1.8–2.0) was determined using the TGem spectrophotometer Plus (Tiangen Biotech, Beijing, China). cDNA was obtained from 1 µg of the total RNA using FastKing kit gDNA Dispelling RT SuperMix (Tiangen Biotech) according to the manufacturer’s protocols. qRT-PCR was performed on a Stratagene Mx3000P system (Agilent Technologies, CA, USA) using SYBR Green chemistry. The 20-µL PCR mixture was prepared based on our previous report (Ndayambaza et al., 2025). Gene-specific primers were designed by NCBI Primer-Blast (**Supplementary Table S1**). All reactions were run in triplicate for each seedling biological replicate, and the expression levels were standardized against the internal control gene *Peuactin*.

### 2.5 Statistical analyses

Relative gene expression was quantified by the 2^−ΔΔCT^ method, with the target expression in the control samples set as 1. Data are expressed as mean ± SE from a minimum of three separate biological replicates. Statistical significance was determined by one-way ANOVA analysis of variance and Duncan’s multiple-range tests as *post-hoc* test via the IBM SPSS Statistics (22.0, Armonk, NY, USA). *P*-value <0.05 was considered to indicate a statistically significant difference. All graphs were drawn on the basis of Origin software (Version 2020, OriginLab; Northampton, MA, USA).

## 3 Results

### 3.1 Isolation and characterization of WOX gene family in *P. euphratica*

The 18 P*. euphratica* WOX genes in the whole genome were ultimately determined, and they were designated as PeuWOX1–PeuWOX18 on the basis of their sequential place in NCBI protein database after obtaining the overlapping proteins. The Pfam domain (PF00046) was retrieved from the *P. euphratica* genome database and duplicate genes were successivetly removed. We identified a collection of 18 protein sequences with lengths varying from 172 (PeuWOX8) to 387 residues (the average length of the proteins was equal to 267.1). By contrast, after we carried out BLASTP analysis, we identified a WOX gene family through Zhang et al.’s protein database; 16 were identified as historical helix-loop-helix-turn-helix (HTH) structures, and these were in common with the findings by Ma et al. because these proteins have the same identical protein numbers as shown in **Supplementary Table S2**. Compared with *A. thaliana*, identical WOX members are found between *P. euphratica* and *A. thaliana* (15) (Cheng, 2022), and original reports of tiling array data show that *P. euphratica* has 18 WOX genes, which is also the case in *P. trichocarp*a (Jia et al., 2025). The deduced WOX proteins (namely, PeuWOX1 to PeuWOX18) were designated based on the chromosomal positions. As shown in **Supplementary Table S2**, the length of their coding sequences (CDSs) ranged from 519 bp (*PeuWOX8*) to 1164 bp (*PeuWOX4*), with an average length of 804.2 bp. This translates into predicted proteins of 172 aa (PeuWOX8) to 387 aa (PeuWOX4), with an average size of 267.1 aa. The theoretical molecular weights differed greatly as well and ranged between 20,021.86 Da (PeuWOX8) and 44,043.72 Da (PeuWOX4), as shown in **Supplementary Table S3**. The estimated isoelectric points (pI) of most proteins ranged from 5.46 to 9.3, and all were less than 10.

### 3.2 Phylogenetic trees, conserved motifs, and gene structure analysis of *PeuWOX* genes

**Figure 1:**
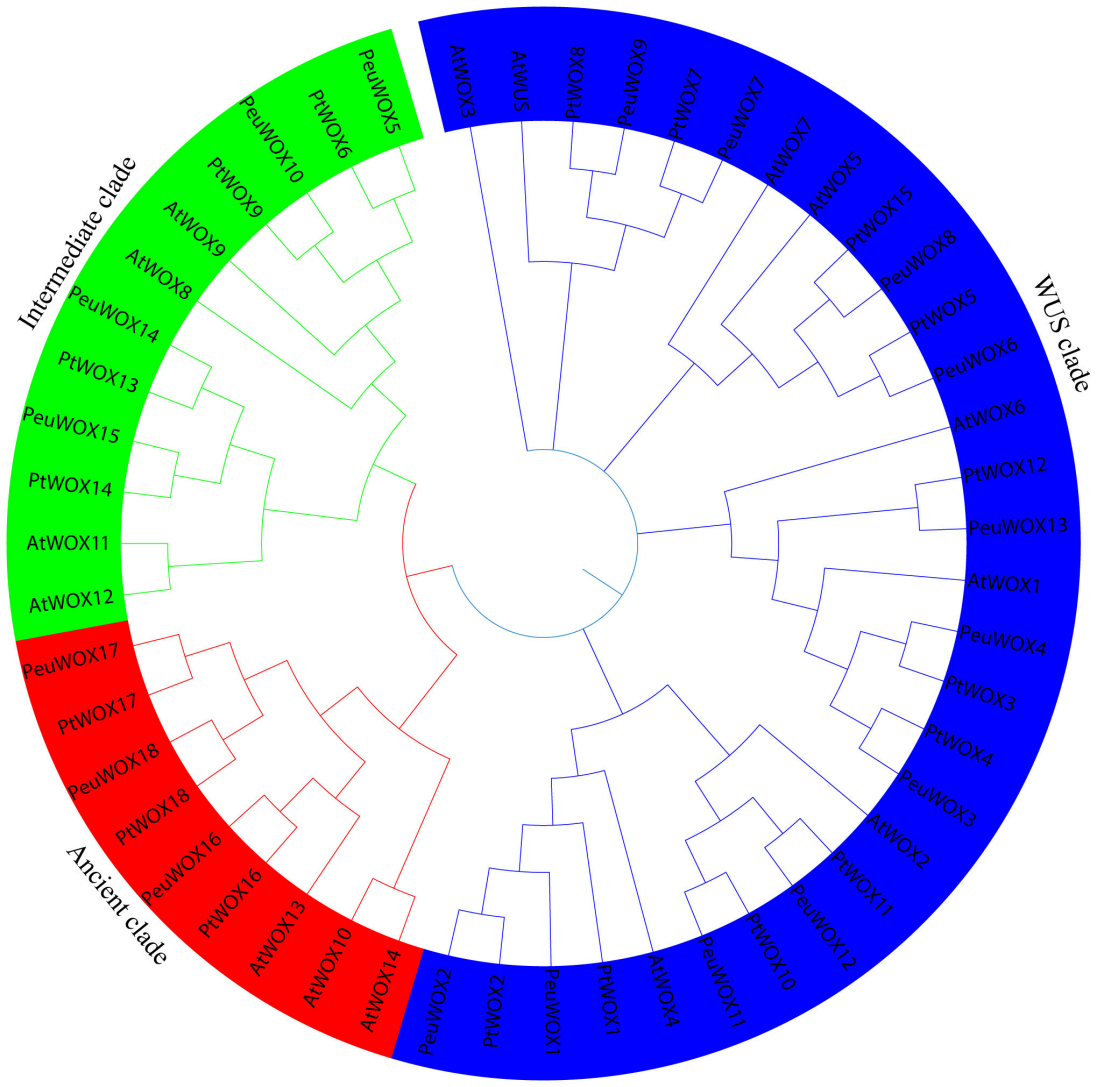
Phylogenetic analysis of WOX proteins: 18 PeuWOX proteins, 18 PtWOX proteins, and 15 AtWOX proteins were aligned using Clustal W. Successively, a phylogenetic tree was generated with MEGA 7 employing the neighbor-joining (NJ) method, supported by 1,000 bootstrap replicates.

In order to clarify the phylogenetic relationships of the WUSCHEL-related homeobox (WOX) transcription factor family, a phylogenetic tree was constructed with this gene family from *A. thaliana* (15), *P. trichocarpa* (18), and *P. euphratica* (18) using the neighbor-joining method (**Figure 1**). The proteins we detected could be divided into three different clades: ancient, intermediate, and modern (WUS). The recent clade was the largest and consisted not only all eight *AtWOXs* but also all 11 from each poplar species. The intermediate and upper clades presented greater conservation, with similar numbers of homologues in each species (four and three members, respectively).

**Figure 2:**
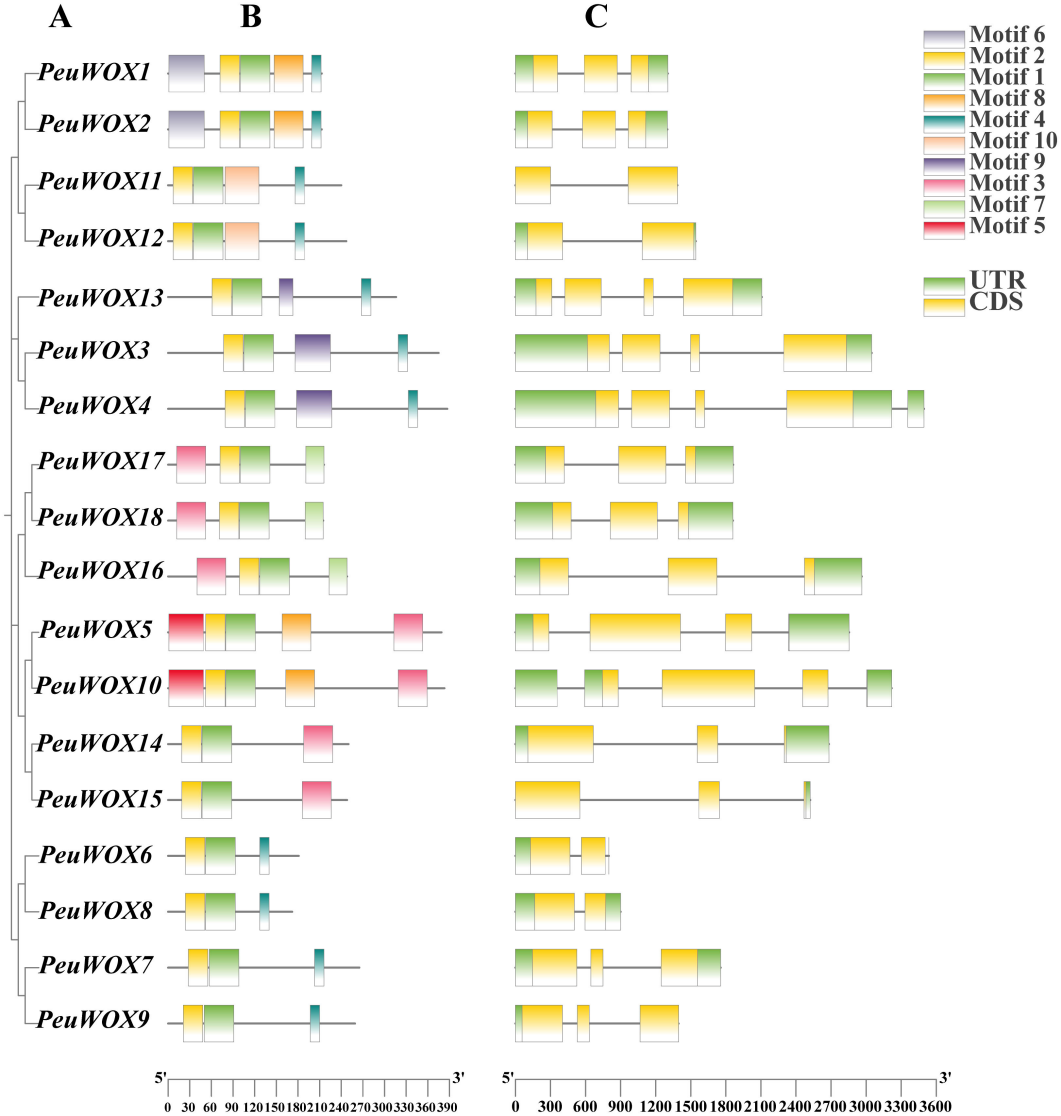
Phylogenetic relationship, gene structure, and motif composition of WOX genes in *P. euphratica*. **(A)** The multiple positions of full-length WOX protein sequences from three species were developed via Clustal W, and a phylogenetic tree was built with MEGA 7 by the neighbor joining (NJ) method with 1,000 bootstrap replicates. The depiction image of conserved motifs was acquired using motif elicitation (MEME) in WOX proteins. The 10-motif composition model in the PeuWOX whole amino acid sequences was primed by MEME.XML through TBtools software II (Toolbox for Biologists v1.130, University of Science and Technology, Wuhan, China). Coding sequence (CDS) and untranslated regions (UTR) and various motifs are represented by boxes of diverse colors. **(B)** Gene structure of WOX gene family analysis. **(C)** Exon/intron structure of the WOX genes. Green and yellow boxes indicate exons, and gray lines represent introns of the WOX genes.

To further understand the conserved domains and gene structure in *PeuWOXs*, we combined motif analysis with phylogenetic tree gene family (**Figure 2A**). The MEME heat map of conserved motifs analysis indicated that there were 10 specific motifs in the PeuWOX family (**Figure 2B**). Motifs 1 and 2 were common among all of the proteins, whereas motif 3 was specific to the ancient/intermediate clades (PeuWOX5, PeuWOX10, PeuWOX14, PeuWOX15, PeuWOX16, PeuWOX17, and PeuWOX18). The other motifs exhibited clade-specific distribution. A comparison of exon–intron organization revealed that closely related PeuWOX proteins are clustered in the same clade and have similar gene structures, including intron numbers (**Figure 2C**). The close relationship between phylogenetic clustering and conservation of gene structures, including motifs and exon–intron organizations, indicates that the evolutionary divisions in *PeuWOX* transcription factor family are supported. Interestingly, all putative genes were found to be multi-exonic with exons and introns.

### 3.3 Cis-acting element analysis in the promoters of the *PeuWOX* genes

**Figure 3:**
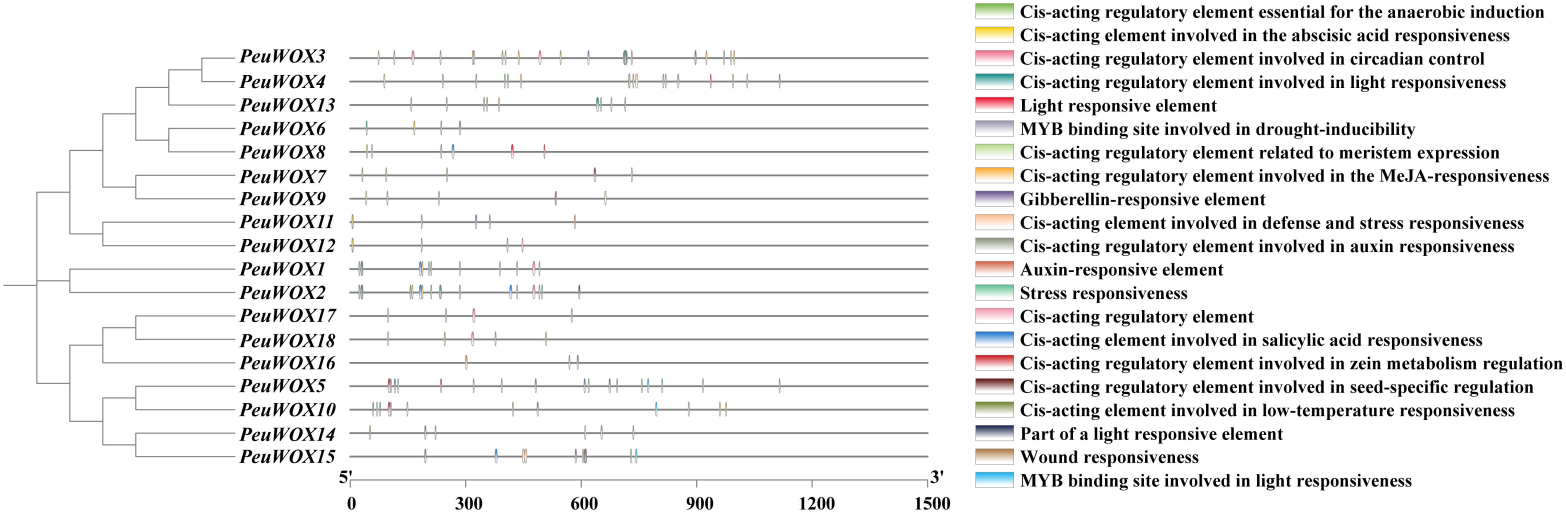
*Cis-*acting elements in the promoter region 1,500-bp upstream of the *PeuWOX* genes were investigated. The numbers and altered colors represent the quantity of *cis-*acting elements. Boxes in various colors exemplify the dispersal of *cis-*acting elements across diverse gene types, which were classified based on their functions: plant growth and development, phytohormone responsive, and abiotic and biotic stresses.

*P. euphratica*, a typical drought- and salt-tolerant species, plays an important role as a woody tree in the afforestation of arid regions and degraded soils. Dissecting the regulatory mechanisms of this tolerance necessitates a comprehensive understanding of its cis-acting elements, which provides crucial insights into their functional roles in stress response. A bioinformatics analysis of the 1,500-bp upstream promoter regions for the *PeuWOX* gene family was conducted via PlantCARE. As shown in **Figure 3**, the promoters consisted of a complicated set of cis-elements as well as core CAAT and TATA boxes. This latter repertoire was significantly enriched for protein components of hormone signaling and stress response. A notable feature is the richness of stress- and hormone-responsive elements, such as ABRE (abscisic acid response), TATC-box (gibberellin response), TCA-element (salicylic acid response), CGTCA-motif (methyl jasmonate response), and AuxRR-core element (auxin response). In addition, the promoters encompassed major development regulators such as CAT-box (meristematic development), circadian elements (circadian control), and MSA-like elements (cell cycle regulation), as demonstrated in **Supplementary Table S4**. The existence of photo-responsive elements was also widely detected. The uniformly complex profile set out in **Figure 3** substantiates that *PeuWOX* genes might be regulated by a variety of hormone signals, environmental stresses, and developmental cues, which further supports their multifaceted functional roles involved in the growth and stress responses of *P. euphratica*.

### 3.4 Chromosomal locations, Ka/Ks ratio, and collinearity analysis of *PeuWOX* genes

**Figure 4:**
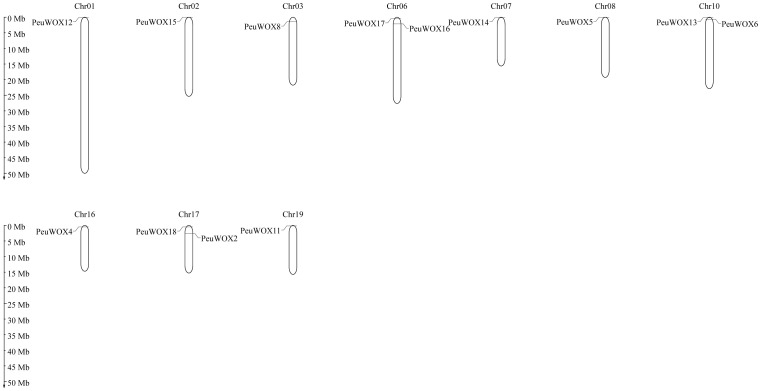
The chromosomal positions of WOX genes in *P. euphratica* species were investigated. In total, 18 *PeuWOX* genes were identified, distributed on chromosomes 1, 2, 3, 6, 7, 8, 10, 16, 17, 18, and 19. The chromosome numbers for each gene are displayed at the top of their respective chromosomes.

The genomic organization of *PeuWOX* genes clustering on chromosomes was analyzed to locate the 18 identified *PeuWOX*s (**Figure 4**). They were located on 19 chromosomes with an uneven distribution—for example, chromosomes 6, 10, and 17 consisted of two genes representing the major linkage groups, respectively. One WOX gene was distributed on chromosomes 1, 2, 3, 7, 8, 16, and 19; no gene resided in chromosomes 5, 12, 13, 14, or 18. This uneven chromosomal dispersal of WOXs is reliable with clarifications in other poplar species (Yang et al., 2025). It is worth mentioning that *PeuWOX1* and *PeuWOX7* were not anchored on any chromosome in the genome assemblies of Zhang et al. and Ma et al., indicating that they reside in the unassembled parts of the genome.

**Figure 5:**
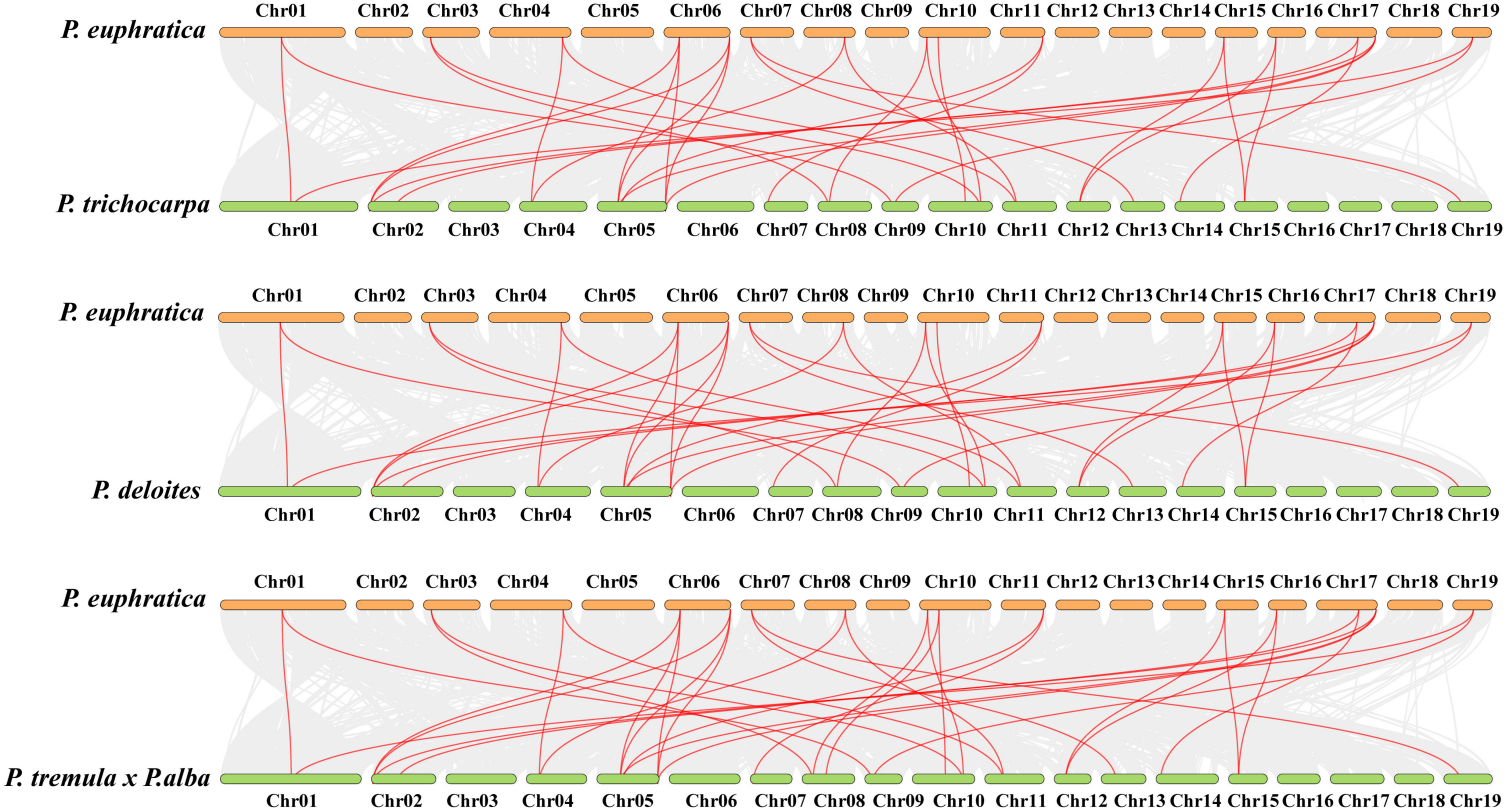
Synteny study of *PeuWOX* and other species. WOX gene collinearity among *P. euphratica* and three descriptive plants (*P. trichocarpa*, *P. deltoides*, and *P. tremula* × *P. alba*) was analyzed using comparative genomics. We investigated the synteny collinearity comparison between *P. euphratica* and three other species. While the red lines in the background designate homozygous WOX gene pairs, the gray lines in the background designate blocks of collinearity within *P. euphratica* and the plants shown.

The selective pressure acted on the *PeuWOX* genes was calculated by the Ka/Ks ratio of nonsynonymous to synonymous substitutions for eight paralogous gene pairs. These pairs (*PeuWOX1/2*, *PeuWOX3/4*, *PeuWOX5/10*, *PeuWOX6/8*, *PeuWOX7/9*, and *PeuWOX11/12*; *PeuWOX14/15* and *PeuWOX17/18*) were thought to arise from whole-genome duplication. A total of four pairs had estimated divergence variance from 17 to 27 MYA. As detailed in **Table 1**, the Ka/Ks ratios were all much lower than 1. In order to evaluate evolutionary conservation, we investigated the synteny of *PeuWOX* genes by MCScanX in TBtool II. A total of 32 syntenic WOX gene pairs were found between the four poplar species (*P. euphratica*, *P. trichocarpa*, *P. deltoides*, and *P. tremula × P. alba*). The abundance of syntenic pairs found in a pairwise manner (**Supplementary Table S5**; **Figure 5**) reveals that the genomic context of WOX family members is extremely conserved among poplar lines.

**Table 1:** Ka/Ks analysis and predictable divergence time (MYA).

Gene pairs	Ka	Ks	Ka_Ks	Time (MYA)
*PeuWOX1/PeuWOX2*	0.036754547	0.248355137	0.147991893	18.92950743
*PeuWOX11/PeuWOX12*	0.144578509	0.225139225	0.642173791	17.16000189
*PeuWOX3/PeuWOX4*	0.098361383	0.251439171	0.391193552	19.16457098
*PeuWOX17/PeuWOX18*	0.059164052	0.306181202	0.19323215	23.33698184
*PeuWOX5/PeuWOX10*	0.090380642	0.366094728	0.246877747	27.90356157
*PeuWOX14/PeuWOX15*	0.077099295	0.294553739	0.261749504	22.45074229
*PeuWOX6/PeuWOX8*	0.073021799	0.312120298	0.23395402	23.78965685
*PeuWOX7/PeuWOX9*	0.077445857	0.168491543	0.459642399	12.84234323

### 3.5 Analysis of the expression pattern of *PeuWOX* genes in various tissues under salt stress

**Figure 6:**
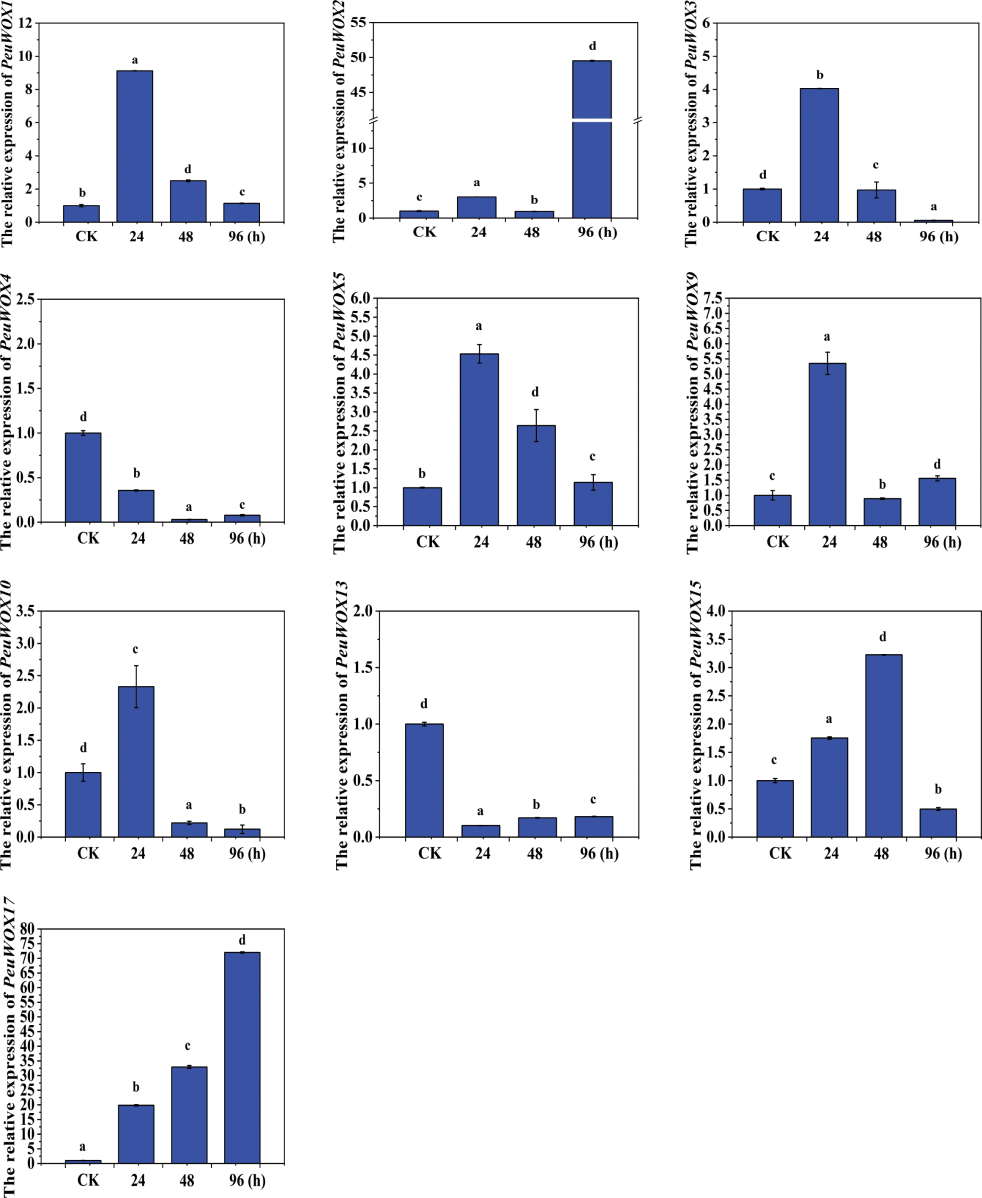
Relative expression analysis of the *PeuWOX* genes in response to stem tissues under salt stress. We validated and quantified the *PeuWOX* expression pattern in stem tissues by analyzing the response of the 10 selected *PeuWOX* genes to NaCl treatment at 24, 48, and 96 h. The control’s relative expression was set to 1. Duncan’s test and one-way ANOVA were used to compare the *PeuWOX* expression relative to the control. Mean ± standard error (SE) was used to exhibit the data. The standard deviations of three replicates are shown by error bars. In this study, three biological repetitions were used, and significant differences were indicated by the letters a, b, c, and d; p-values (p ≤ 0.05) were determined using the least significant difference (LSD) test.

To examine whether salt stress response was involved in the *PeuWOX* genes, using the qRT-PCR method, we examined the expression pattern of 10 selected *PeuWOX* genes in three tissues/organs. In stem organs under salt stress (200 mM NaCl treatment), the expression levels were dynamically responded over time (24, 48, and 96 h), which illustrates that they are involved in salt adaptation and adventitious root control. Most of the *PeuWOX* genes were expressed strongly over time, and this was an indicator that the family might have evolved through functional divergence. The *PeuWOX*2 and *PeuWOX*17 genes were highly induced during long-term salt stress, and the point of maximum expression was at 96 h, indicating that they are major regulators of late response to salinity and cell reprogramming when cells are subjected to salt stress conditions. The *PeuWOX*3 and *PeuWOX*5 genes both exhibited early upregulation (24–48 h) and downregulation (96 h), which highlights their potential involvement in the early activation of stress-responsive developmental events, including the initiation of adventitious rooting or stem tissue remodeling. Particularly, the expression of *PeuWOX*9 and *PeuWOX*10 genes was significantly increased at 48 h, which could have been involved in the retention of meristematic activity and cell proliferative potential under osmotic stress. These genes suggest the same function of WOX11/12 homologs which were previously found to be stimulated by salinity, resulting in root initiation and growth in Populus and Arabidopsis species (Liu et al., 2022; Wang et al., 2021). Nevertheless, the *PeuWOX*4, *PeuWOX*10, and *PeuWOX*13 genes were repressed or temporarily expressed, which indicated that these genes could not be activated in order to modulate energy and develop signal distribution toward adaptive growth. In contrast, we observed an exceptional improvement of *PeuWOX*17, especially at 96 h, indicating that it is a potential late-stage regulator governing tissue differentiation and stress recovery (**Figure 6**) (Li et al., 2022; Minh-Thu et al., 2018). Taken together, these results suggest that *PeuWOX* genes play a crucial role in a highly synchronized, time-dependent regulatory system of salt stress that controls growth arrest and regenerative/adaptive reactions in stems. This transcriptional plasticity could be the reason behind the remarkable survival of this species in saline and arid areas that could increase the observed meristem activation via WOX to generate adventitious roots and subsequent long-term survival.

**Figure 7:**
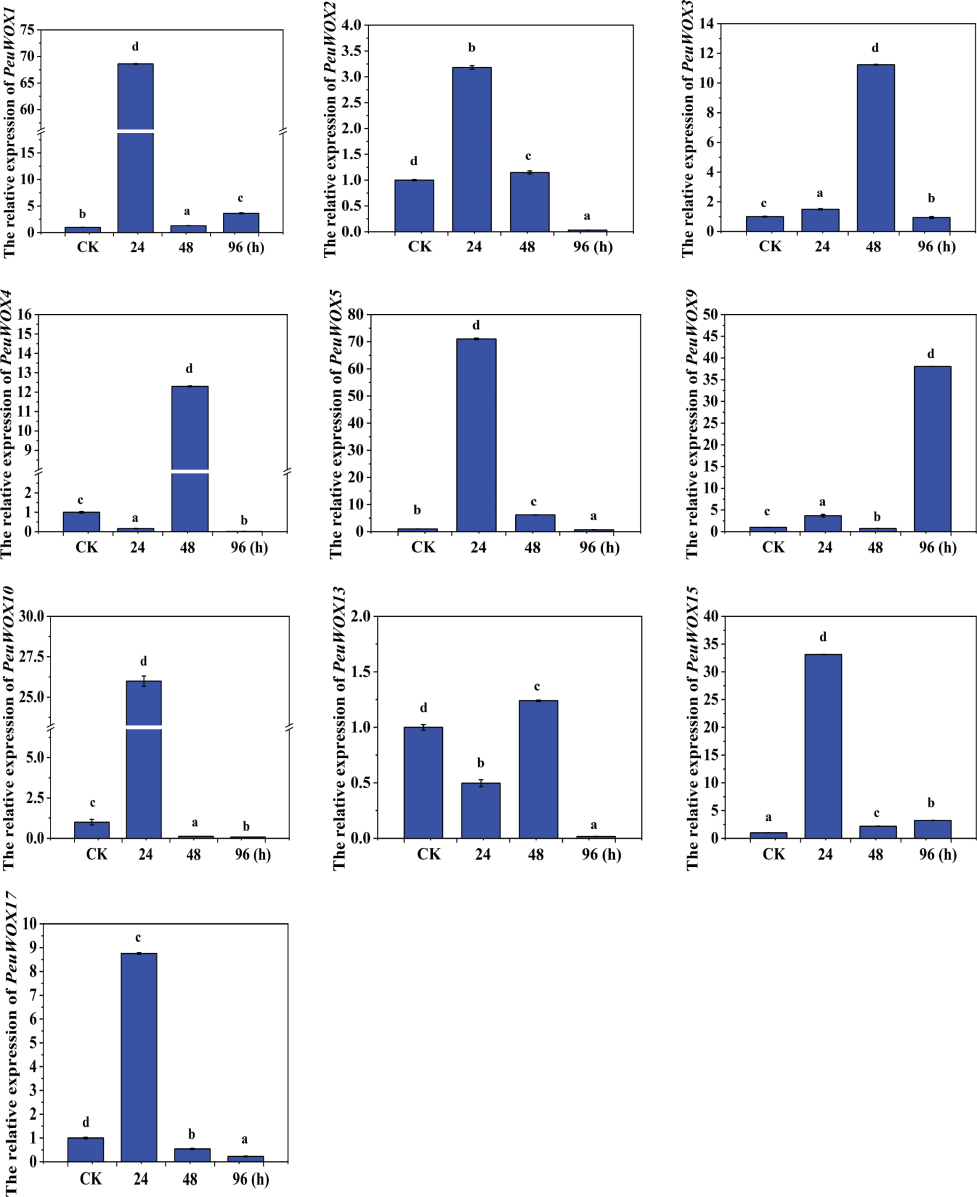
The relative expression analysis of the *PeuWOX* genes in response to root tissues under salt stress. We validated and quantified the *PeuWOX* expression pattern in root tissues by analyzing the response of the 10 selected *PeuWOX* genes to NaCl treatment at 24, 48, and 96h. The control’s relative expression was set to 1. Duncan’s test and one-way ANOVA were used to compare the *PeuWOX*’s expression relative to the control. Mean ± standard error (SE) was used to exhibit the data. The standard deviations of three replicates are shown by error bars. In this study, three biological replications were used, and significant differences were designated by the letters a, b, c, and d; p-values (p ≤ 0.05) were determined using the least significant difference (LSD) test.

In root tissues under salt stress, the *PeuWOX* gene family expression pattern in root organs after treatment was observed to be different and dynamic at different periods (including control and 24, 48, and 96 h) under 200 mM NaCl treatment (**Figure 7**). Such observed trend means that the WOX genes could be at the center of salt stress-induced root adaptation. There was an observed general association in which most of the *PeuWOX* genes were upregulated at an early stage (powerfully at least up to 48 h following NaCl exposure) and then downregulated under control at the later period of 96 h, indicating that WOX signaling is urgent to initial perception of the stress and adventitious root initiation. Specifically, the expression pattern of *PeuWOX1*, *PeuWOX4*, *PeuWOX5*, *PeuWOX10*, and *PeuWOX13* genes was exhibited with a significant peak at 48 h (**Figure 7**). The family members that were highly induced in such cluster were *PeuWOX5* and *PeuWOX10* genes, indicating that they were likely to be involved in processes like cell proliferation, reactivation of meristem activity, or adventitious root formation in response to salt stress conditions. These findings are consonant and are backed by earlier publications (Xu et al., 2025). The previous studies have demonstrated the role of WOX11/12 genes in model species as Arabidopsis thaliana and Populus spp. in the process of controlling the decision of cell fate, *de novo* root organogenesis, and regardless of morphogenetic-like genetic processes has also been demonstrated under abiotic stress conditions (Liu et al., 2014a; Liu et al., 2014b). Furthermore, the initial and short-lived expression of genes, like *PeuWOX2*, *PeuWOX3*, and *PeuWOX9* between 24 and 48 h suggests that they play a role in the priming phase of the root primordia formation in addition to mediate/modulating cascades of auxin signaling. This hypothetical role is in line with the observation that WOXs belong to a family of transcription factors, which often interact downstream of ARFs in the stimulation of both lateral and adventitious root initiation.

Conspicuously, the *PeuWOX17* gene displayed a distinct and delayed expression pattern level with an evident enhancement at 24 h and a spectacular decrease at 48 and 96 h post-treatment, and therefore it can also serve as an early signal integrator in reaction to salt-induced osmotic distress rather than being a long-term active protein. This type of transient upregulation is also in agreement with the WOX-like activity of the transient activation of transcriptional networks responsive to stress before developmental adaptation as proposed previously (Liu et al., 2014a; Zhang et al., 2022, 2010). In contrast, the *PeuWOX9* and *PeuWOX13* genes showed a secondary peak at 48 h, indicating that these genes are involved in the strengthening of adaptive root remodeling after cessation of primary stress signaling (as depicted in **Figure 7**). All of these findings indicate that the *PeuWOX* genes in the roots are temporally controlled under salt conditions that mediate early developmental and hormonal responses in the plasticity and integrity of the root structure. Interestingly, the upregulation of *PeuWOX5* and *PeuWOX10* genes is rapid at 24 h, which suggests that these genes play a significant role in facilitating the adventitious regeneration of roots under salt stress. This could be one of the factors that led to this desert species being able to flourish and even grow well in strong-saline arid soil regions. The discovered transcriptional plasticity of the WOX family underscores their significance in the evolution of stress tolerance among perennial woody species.

**Figure 8:**
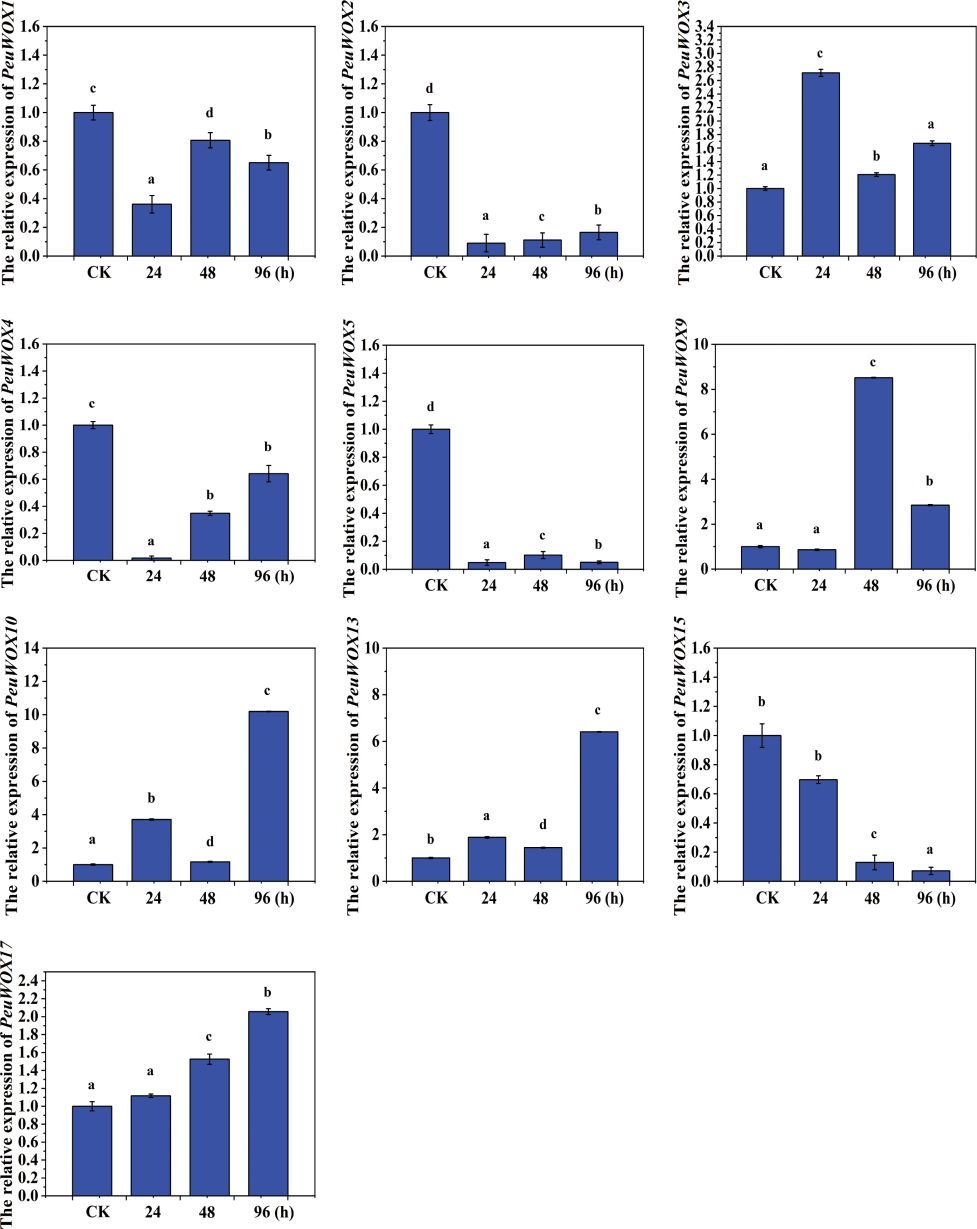
Expression pattern analysis of *PeuWOX* genes in leaf tissues under salt stress. To validate and quantify the pattern of the *PeuWOX* expression in leaf tissues, 10 selected *PeuWOX* genes were analyzed in terms of the response to NaCl treatment at 0 (control), 24, 48, and 96 h. The relative expression of the control was adjusted to 1. To compare the expression of *PeuWOX* genes, Duncan’s test and one-way ANOVA were conducted. The data are exhibited as mean ± standard error (SE). Error bars demonstrate the standard deviations of three replicates. This study utilized three biological replications and had assigned significant differences with letters a, b, c, and d and p-values (p ≤ 0.05) through least significant difference (LSD) test.

In leaf tissues under salt stress, the expression pattern levels of the 10 genes of interest (PeuWOX) of poplar leaves under salt stress exhibit diverse temporal expressions that characterize genes relating to the initial shock of the stress, intermediate response, and long-term adaptation. Such a time distance symbolizes functional divergence across the WOX family in reacting to soil salinity, and some of the members might be engaged in stress sensitivity, as opposed to tolerance. As an example, the expression pattern levels of the *PeuWOX1*, *PeuWOX2*, *PeuWOX4*, *PeuWOX5*, *PeuWOX9*, and *PeuWOX15* genes under control (CK) conditions are vigorously and rapidly suppressed by treatment with salt after 24 h, indicating that they exhibit a relatively high constitutive expression, as it is reflected in a significant decrease (as illustrated in **Figure 8**). This acute deactivation suggests that their coded functions, that are likely to be associated with normal leaf organogenesis, vascular organization, and meristem homeostasis (Kucukoglu et al., 2017; Vandenbussche et al., 2009), cannot be energetically apt to immediate osmotic and ionic variation that is needed to survive. Even though transcriptional recovery is partially recovered after 48 and 96 h in the *PeuWOX1*, *PeuWOX4*, and *PeuWOX5* genes, there is no long-term recovery, and in fact, the *PeuWOX2*, *PeuWOX9*, and *PeuWOX15* genes are permanently (or augmented) suppressed as demonstrated in **Figure 8**. The fact that these pro-growth genes were further repressed is a sign of a tradeoff between cellular growth and defense, an ancient mechanism of adaptation to stress that underlies growth inhibition in the case of soil salinity (Skirycz and Inzé, 2010).

On the other hand, a number of *PeuWOX* genes have high inducible expression patterns. The transient increase of the *PeuWOX3* gene peaking at 48 h and then decreasing also agrees with the fact that it has a role in restoring developmental homeostasis after the initial osmotic shock. Conversely, the *PeuWOX17* gene demonstrates a slow, continuous accumulation with duration of stress and can therefore be involved in long-term acclimation, possibly the modulation of hormone signaling or cellular plasticity. Interestingly, it is found that the *PeuWOX10* and *PeuWOX13* genes are drastically induced in salt stress at the late (96 h) time point (**Figure 8**). The trend conforms to their previous functions in their capacity as adapters of adverse abiotic stress (Zhou et al., 2024). They are robust and extremely induced during their late stages that put them as the control mechanism of long-term protective mechanisms comprising antioxidant protection, osmotic regulation, and tissue remodeling. Therefore, the *PeuWOX10*, *PeuWOX13*, and *PeuWOX17* genes may preferentially play a significant role to induce long-term adaptive change in the leaves of desert poplar species under salt stress

### 3.6 Analysis of the expression patterns of *PeuWOX* genes in various organs under drought stress

**Figure 9:**
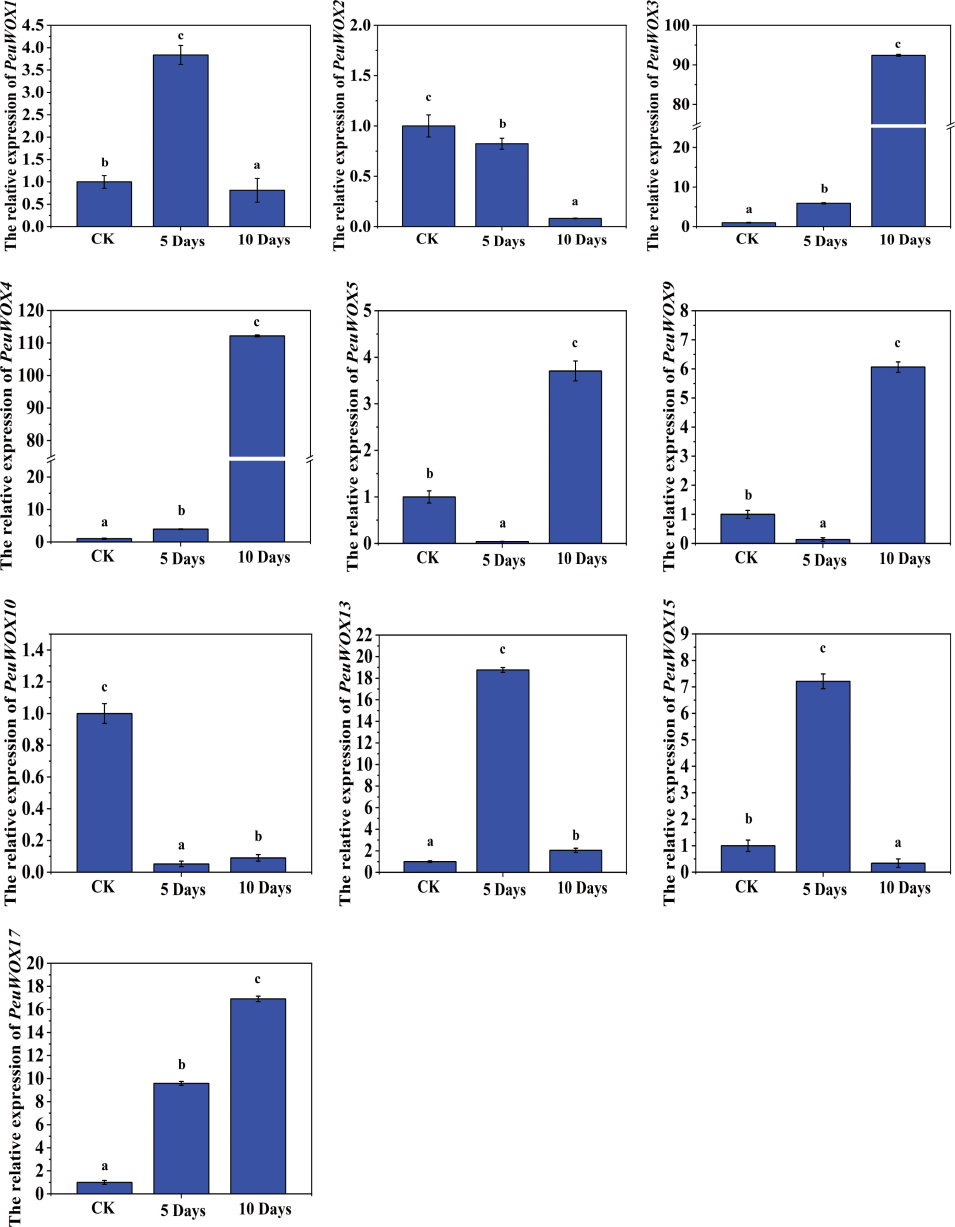
Expression pattern analysis of *PeuWOX* genes in stem tissues under drought stress. The expression pattern of the 10 selected *PeuWOX* genes in P. euphratica stems under drought stress conditions. The x-axis shows the time after the onset of stress treatments. The standard deviations of the data are exhibited by the error bars. Three biological replicates were used. Three types of drought were studied along with the related soil volumetric water content (soil-VWC): mild drought (31% ± 1% soil-VWC, 5 days), moderate drought (21% ± 1% soil-VWC, 10 days), and control (41% ± 1% soil-VWC). In the control group, the relative expression was set to 1. Using Duncan’s test and one-way ANOVA, the relative expression of *PeuWOXs* was compared to the control. Information was demonstrated as the mean ± standard error (SE). Three replicates’ standard deviations are shown by error bars. The stress treatment groups indicated by the letters a, b, c, and d indicated a significant difference.

The expression pattern levels of the *PeuWOX* genes in the stem organs have revealed that individual WOX genes are responding dynamically and responsively to growth and developmental plasticity under drought stress. All of these exposures to drought had a profound influence on the expression of the majority of the PeuWOX genes, which suggests that the WOX transcription factors play a vital role of signaling cascades in regulating stem development, tissue repair, and AR development under conditions of water scarcity. Many WOX genes such as *PeuWOX1*, *PeuWOX3*, *PeuWOX4*, *PeuWOX5*, *PeuWOX9*, *PeuWOX13*, and *PeuWOX17* genes were also highly upregulated following the induction of drought though the time peaks varied (**Figure 9**). Even greater increments in the expression pattern levels were observed in the *PeuWOX3*, *PeuWOX4*, and *PeuWOX5* genes at the later time points and at 10 days, respectively, which suggest that these genes play an important role in growth maintenance and the integrity of stem tissue during long-term drought stress. This coincides with other studies which found that WOX4 and WOX5 orthologs retain vascular cambium activity and offer meristematic support during abiotic stress (Yang et al., 2023) and, thus, can grow further when exposed to a water loss condition (Rasheed et al., 2024; Tvorogova et al., 2021). Conversely, the *PeuWOX1* and *PeuWOX9* genes were dramatically induced during 10-day drought stress, which can be related to their potential role in cell fate reprogramming and secondary growth process activation as WOX1-type genes are considered to have regulatory activities in organ boundary development and tissue differentiation (Li et al., 2024). In contrast, genes such as *PeuWOX2*, *PeuWOX10*, and *PeuWOX13* have been found to be expressed early, with an increase in their expression levels at 5 days prior to a reduction at 10 days, suggesting their role in early drought signaling response potentially to stress sensing to initiate adaptive developmental reprogramming. An early hormonal response might be linked with such a temporary induction, especially with auxin and ABA crosstalk since WOX genes have been demonstrated to be direct downstream targets of an auxin-mediated process (Xu et al., 2023). The *PeuWOX17* gene exhibited a gradual accumulation of drought duration, which persisted to accumulate at 5 and 10 days, which is consistent with the fact that it is a long-term stress tolerance factor that preserves the performance of the stem cell and increases tissue resistance to stressful environments as reported (Tang et al., 2022). The members of the P. euphratica WOX gene family together change in time and functionality in response to drought stress. The early-responsive genes (*PeuWOX2* and *PeuWOX10*) could mediate the carbon dioxide early signal transmission and perception of stress, whereas the later-responsive genes (*PeuWOX3*, *PeuWOX4*, *PeuWOX5*, *PeuWOX9*, and *PeuWOX17* genes) would be involved in developmental changes such as vascular tissue strengthening during the adventitious root occurrence and root loss to replenish the stem regeneration (**Figure 9**). Such alterations in gene expression are probably the factors that make P. euphratica experience an unprecedented level of drought resistance and thus be able to have a recovery of growth despite the surrounding extreme and environment. Therefore, the concerted regulation of WOX genes highlights the evolutionary significance and their contribution to structural and physiological plasticity to severe water stress.

**Figure 10:**
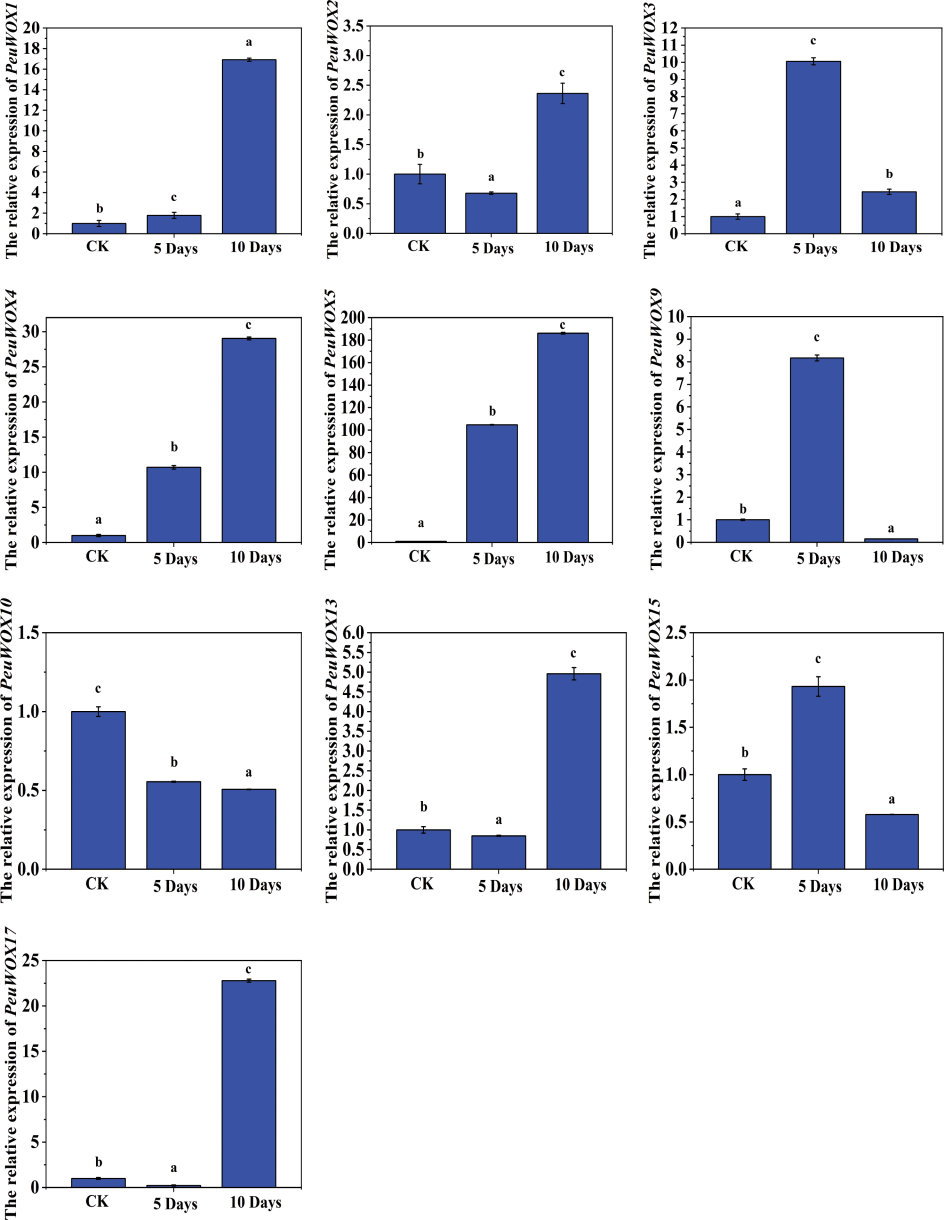
Expression pattern analysis of *PeuWOX* genes in root tissues under drought stress. The expression pattern of the 10 selected *PeuWOX* genes, in P. euphratica roots under drought stress conditions. The x-axis shows time after the onset of stress treatments. The standard deviations of the data are exhibited by the error bars. Three biological replicates were used. Three types of drought were studied along with the related soil volumetric water content (soil-VWC): mild drought (31% ± 1% soil-VWC, 5 days), moderate drought (21% ± 1% soil-VWC, 10 days), and control (41% ± 1% soil-VWC). In the control group, the relative expression was set to 1. Using Duncan’s test and one-way ANOVA, the relative expression of *PeuWOX*s was compared to the control. Information was demonstrated as mean ± standard error (SE). Three replicates’ standard deviations are shown by error bars. The stress treatment groups indicated by the letters a, b, c, and d indicated a significant difference.

The expression pattern levels of *PeuWOX* (control and 5 and 10 days of water deficit) under drought stress show that drought can functionally regulate *PeuWOX* transcription factor at the level of a single gene and is also time dependent in root tissues. These data show that the WOX genes play diverse but interactive roles in the process of controlling root developmental plasticity, tissues, and adaptation to drought stress process that is needed to confer P. euphratica with its remarkable drought tolerance in desert environments. Overall, most of the *PeuWOX* genes (*PeuWOX1*, *PeuWOX3*, *PeuWOX4*, *PeuWOX5*, *PeuWOX9*, *PeuWOX13*, and *PeuWOX17*) were highly induced by drought and reached their peak at either 5 or 10 days after treatment (**Figure 10**). The continuous upregulation of the *PeuWOX1*, *PeuWOX5*, *PeuWOX9*, and *PeuWOX17* genes with a peak of expression level on day 10 indicated that these genes might be associated with the intention of keeping the activity of the roots and the conservation of the meristematic cell identity under the conditions of prolonged drought. This sustained upregulation is also in line with the finding that WOX5/WOX7 homologs have roles in the retention of root apical meristem and subsequent root initiation in abiotic stress, as reported in previous studies (Willig et al., 2024; Xin et al., 2024). In contrast, the *PeuWOX3*, *PeuWOX10*, and *PeuWOX15* genes were found to be maximally expressed at 5 days and reduced at 10 days, suggesting that these three genes belong to early drought perception and signaling. These immediate-response genes can play a role in early stress response through auxin redistribution mechanism or ABA-mediated mechanism because WOX transcription factors are able to interact with hormonal signals to mediate adaptive developmental processes (Hou et al., 2021). As an example, the induction of PeuWOX10 gene at an early stage would prefer the formation of lateral roots that would enhance water uptake as typical drought defense in Populus species (Rasheed et al., 2024; Tang et al., 2022).

Notably, the *PeuWOX4* and *PeuWOX5* genes were the two most significantly increased in their activities, particularly after 10 days of drought treatment, indicating their central roles in the process of tissue differentiation and xylem formation (**Figure 10**). These functions are valuable in the maximization of water transportation in reaction to the decrease of water supply. AtWOX4 in Arabidopsis thaliana serves equivalent functions by stimulating cambial activity and the formation of vascular tissues during stress (von der Mark et al., 2024). In addition, both of the *PeuWOX13* and *PeuWOX17* genes were significantly expressed under the late drought condition, which is in line with their possible role in stress memory formation and regulation of the root system architecture (through mediating stress-induced organ regeneration) as reported in previous studies (Li et al., 2022). Taken together, these findings highlight the fact that under drought conditions, the *PeuWOX* genes in root tissues are temporally differentially expressed due to the action of specific regulatory signals governing the stress signal, root growth, and meristem activity. Initial detection of drought and hormonal regulation may be related to early-responsive WOX genes and later structural reinforcement and developmental strength during extended water scarcity conditions. This transcriptional plasticity should be included in the survival plan developed by *P. euphratica* in arid desert environments, in which root development and vascular differentiation may be able to react rapidly to decreased water levels.

**Figure 11:**
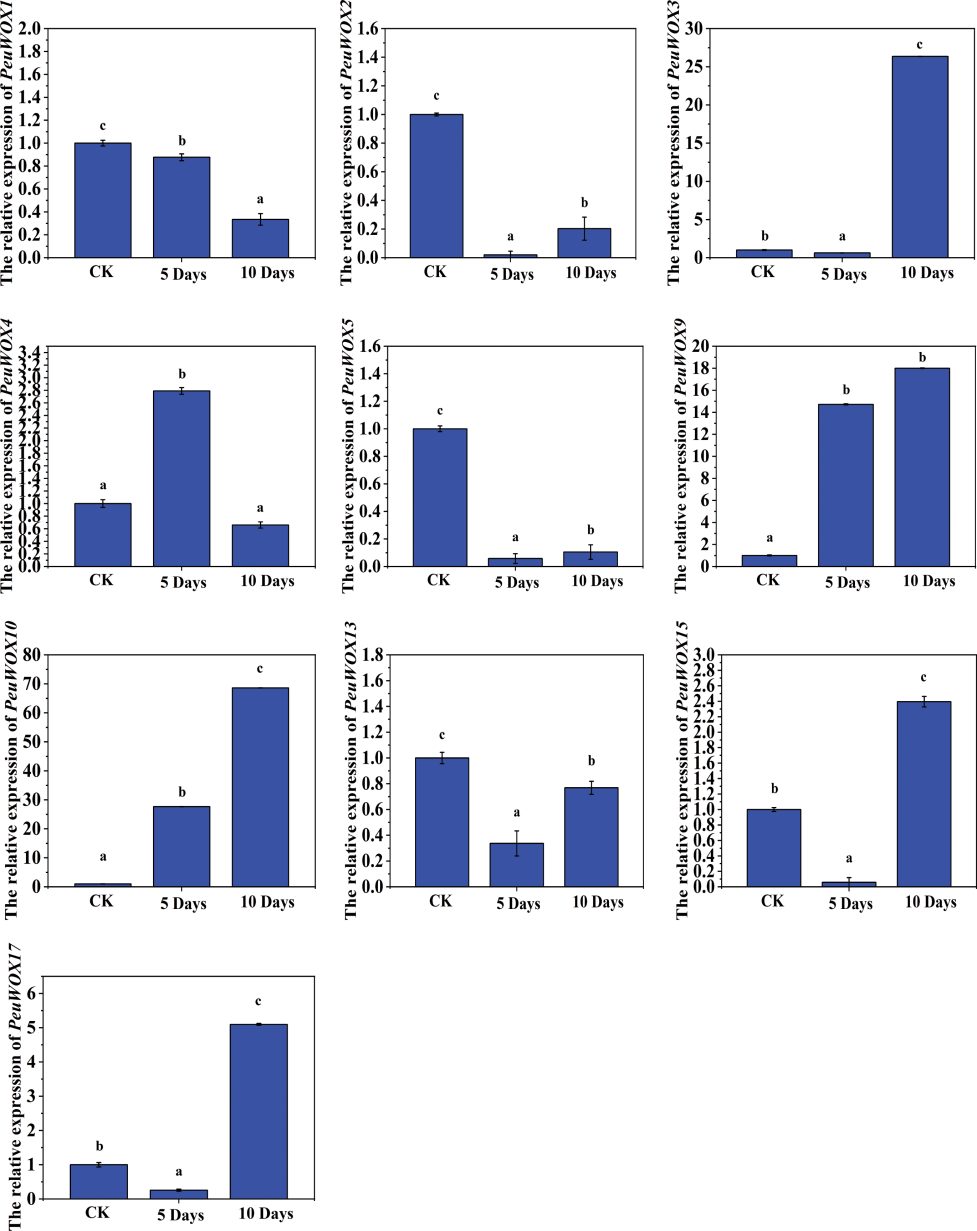
Expression pattern analysis of *PeuWOX* genes in leaf tissues under drought stress. The expression pattern of the 10 selected *PeuWOX* genes in P. euphratica leaves under drought stress conditions. The x-axis shows time after the onset of stress treatments. The standard deviations of the data are exhibited by the error bars. Three biological replicates were used. Three types of drought were studied along with the related soil volumetric water content (soil-VWC): mild drought (31% ± 1% soil-VWC, 5 days), moderate drought (21% ± 1% soil-VWC, 10 days), and control (41% ± 1% soil-VWC). In the control group, the relative expression was set to 1. Using Duncan’s test and one-way ANOVA, the relative expression of *PeuWOXs* was compared to the control. Information was demonstrated as mean ± standard error (SE). Three replicates’ standard deviations are shown by error bars. The stress treatment groups indicated by the letters a, b, c, and d indicated a significant difference.

In order to understand the dynamics of the expression of the *PeuWOX* genes in greater detail, we conducted a qRT-PCR analysis of leaf tissues subjected to gradual drought stress. The findings showed that the expression patterns were different in terms of pronounced downregulation or sharp upregulation with time. In well-watered (CK) conditions, a number of *PeuWOX* genes (*PeuWOX1*, *PeuWOX2*, *PeuWOX4*, *PeuWOX5*, *PeuWOX13*, and *PeuWOX15*) were highly expressed in the leaf base tissues, which is in line with their potential functions of leaf development (**Figure 11**). These genes were downregulated acutely (in 5 days of stress) when exposed to drought. This quick transcriptional inhibition implies strategic early termination of developmental programs which may help in drought avoidance with the minimization of the transpirational surface area and termination of expansive development.

On the other hand, the acute upregulation of *PeuWOX9*, *PeuWOX10*, *PeuWOX15*, and *PeuWOX17* genes occurred after 10 days of drought treatment. The strongest response was recorded in the *PeuWOX10* gene with an increase in and progressive upregulation (**Figure 11**). This tendency means that it plays a significant regulatory role in the process of adapting to long-term drought, potentially by the activation of cellular protection and dedifferentiation pathways (Golldack et al., 2014; Muhammad Tajo et al., 2022). The *PeuWOX3* gene exhibited a low activity with occasional surge at day 5 and then at day 10 showed a high expression, which may reflect the possible functions of sensing stress or subsequent morphological adaptation. Induction of the *PeuWOX9* gene occurred progressively, which is in line with a role in cellular competency in stress. The *PeuWOX15* and *PeuWOX17* genes were suppressed-then-induced and implied as having a role in second adaptive adjustments and fine-tuning of hormonal crosstalk under the long-term water deficit condition (Rosso et al., 2023). The gene of *PeuWOX4* was induced in the short term but fell over time under stress.

These dynamics of expression together demonstrate a coordinated transcriptomic rearrangement of the leaves during drought stress, where the developmental WOX genes are suppressed at the expense of stress-related ones which are induced sequentially in *PeuWOXs*. These results suggest a hypothesis where drought adaptation includes the adaptation of protecting rather than growing. It is also worth mentioning that particular members of the *PeuWOX* family (*PeuWOX3*, *PeuWOX9*, PeuWOX10, and *PeuWOX17* genes) also seem to be essential in long-term survival in water stress, which means that they might be involved in the regulation of adaptive and protective responses.

## 4 Discussion

The WOX transcription factor family plays critical roles in plant growth and development, such as embryogenesis and organ formation (Dolzblasz et al., 2016; Fambrini et al., 2022). Although their roles are well defined in herbaceous models such as *Arabidopsis thaliana*, their role in some stress-tolerant woody perennial trees, especially in harsh environments, has not been studied. *Populus euphratica*, which has been known to be very resilient to drought and salinity (Chen et al., 2015; Ma et al., 2013), is a good system to study the role the WOX family plays in the adaptation of trees to abiotic stresses. Here we were able to identify 18 *PeuWOX* genes and systematically analyze their phylogeny, genome characteristics, and stress-responsive expression. We have found that not only is the *PeuWOX* family conserved in its developmental functions but also it probably co-opted into a complex regulatory system that exerts fine control over growth and stress responses, a theme that has been gaining more and more importance in woody plants. A phylogenetic study placed the 18 *PeuWOX* proteins into three canonical clades—modern (WUS), intermediate, and ancient (**Figure 1**), with proportions equal to that in *P. trichocarpa* (Hou et al., 2021; Wu et al., 2019) and apple (Cheng, 2022) but larger than the 15 representatives in *A. thaliana* (van der Graaff et al., 2009). It is a similar growth to other woody species and could be a foundation to the complexity of development and adaptability to stress of perennial plants (Zhou et al., 2024). The well-preserved gene structure and motifs (motifs 1 and 2) in each clade is evidence of functional divergence in line with phylogenetic conservation (**Figure 2**), presumably organizing a homeodomain essential for binding to DNA (van der Graaff et al., 2009). It is worth noting that the genomic structure of the *PeuWOX* genes, with their asymmetrical chromosomal distribution (**Figure 3**) and wide synteny with other *Populus* species (**Figure 4**), and the Ka/Ks ratios, much lower than 1 (**Table 1**), indicate that the WOX family has experienced intense purifying selection after instances of whole-genome duplication. This evolutionary conservation highlights the basic and indispensable positions of WOX genes in *Populus* species.

Another important observation in our promoter analysis was that stress-/hormone-responsive cis-elements such as ABRE (ABA response), CGTCA-motif (MeJA response), and AuxRR-core (auxin response) were highly enriched, as illustrated in **Figure 5**. The identification of developmental elements such as CAT-box also supports their well-known function in meristem maintenance and organogenesis (Chaudhary et al., 2022). The result offers a direct molecular correlation between the signals of environmental stress and the transcriptional regulation of the genes of *PeuWOX*. The existence of these factors indicates that the expression of *PeuWOX* is regulated by complex hormonal crosstalk, a mechanism that is central to the integration of stress perception and development reprogramming, which is a new paradigm in the role of WOX in the abiotic stress of woody tree species (Li et al., 2022; van der Graaff et al., 2009).

The expression pattern levels of our analysis under drought and salt stress indicated that the patterns of the response of the PeuWOX family is dynamic, a tissue-specific and a temporally coordinated response, emphasizing the possible roles of the family in stress adaptation—for instance, the early and intense induction of *PeuWOX5* and *PeuWOX10* (homologs of *AtWOX11/12*) at 48 h of salt stress in roots (**Figure 7**) and the subsequent activation of adaptive programs, including adventitious root development to promote water and nutrient uptake, indicate an immediate role of these genes in regulating adaptive programs in roots. It is consistent with the preserved role of WOX11/12 clade members to induce cell fate transitions and de novo organogenesis during stress in diverse species, such as poplar species (Liu et al., 2021). These genes were subsequently downregulated at 96 h which could be an accurate metabolic regulation to avoid being overgrown at salt stress conditions (**Figure 7**). We found a very specific strategy of time in the stem tissues under drought conditions—for example, genes such as *PeuWOX4* and *PeuWOX5* were demonstrated to be upregulated in the first place, at 10 days (**Figure 9**), when they are known to have their orthologous roles in sustaining vascular cambium activity and secondary growth (Etchells et al., 2013; Kucukoglu et al., 2017). This chronic induction probably facilitates the maintenance of vascular tissues and stem integrity. In the same way, the late induction of the *PeuWOX17* gene’s strong response to salt stress (96 h) implies a position in long-term stem tissue remodeling and re-establishing of stress (**Figure 6**), similar to the functions of the WOX11 subfamily in other species (Lu et al., 2023).

The expression pattern levels in the tissues of the leaves showed an interesting tradeoff approach, which is a core feature of stress accumulation. The control conditions expressed developmental genes including *PeuWOX1*, *PeuWOX2*, *PeuWOX4*, and *PeuWOX5* but acutely repressed the genes when the organism was exposed to salt stress. This occurrence of rapid transcriptional repression is probably an energy-saving mechanism, which strategically interrupts the broad leaf development process to reduce the transcriptional water loss (**Figure 8**). On the other hand, genes related to salt stress such as *PeuWOX9*, *PeuWOX10*, and *PeuWOX17* were greatly expressed in the case of prolonged stress. The *PeuWOX10* gene, which shared the same expression to the stress-sensitive clade of WOX9 (Zhang et al., 2023b; Zhu et al., 2014), is induced progressively and strongly, indicating its value as a global regulator, activating cellular protection and dedifferentiation networks to survive over the long term of salt stress. This developmental-to-protective transcriptional cascade change is a good example of how the WOX genes can facilitate a growth-to-defense transition which is emphasized in recent reviews on the topic of woody plant responses to stressors (Zhou et al., 2024). Conversely, the survival strategy is a potent representation of the leaf expression in *PeuWOX* under drought stress—for instance, the expression levels of regulation of developmental PeuWOX genes (*PeuWOX1*, *PeuWOX2*, *PeuWOX4*, *PeuWOX5*, *PeuWOX13*, and *PeuWOX15*) in 5 days is a strategic termination of leaf expansion programs in order to save water. On the other hand, the drastic increase of the expression levels of *PeuWOX9*, *PeuWOX10*, and *PeuWOX17* genes in 10 days indicates the transition to an adaptive and protective state (**Figure 11**). *The PeuWOX10* gene shows a rapid induction under drought stress, which is the same stress-responsive WOX13 clade member (Dai et al., 2023). This is particularly interesting and speaks in favor of its hypothetical role as the overall regulator of a long-term drought acclimation process, potentially via the promotion of cellular detoxification and tissue remodeling transcriptional programs (Zhou et al., 2024).

To sum up, we have presented an integrated analysis that allows us to propose a model in which the *PeuWOX* family of P. euphratica is the most important regulatory node that links developmental plasticity with perception of stress. Certain cells are deployed in a tissue- and time-specific way to perform adaptive functions: early responder genes, such as *PeuWOX5*/*PeuWOX10*, trigger root system remodeling, late responder genes, such as *PeuWOX4*/*PeuWOX17*, may improve stem and vascular tissue; and a specialized gene (*PeuWOX10*) upregulates in the leaves to coordinate long-term cellular defenses. This functional division of labor of a preserved transcription factor family probably forms one of the key elements of abiotic stress resilience as an unusual nature of P. euphratica. The study provides a crucial background to future functional research involving genetic modification or gene editing (CRISPR-Cas9) to confirm the functions of candidate genes such as *PeuWOX4*, *PeuWOX5*, *PeuWOX10*, and *PeuWOX17*. Finally, the current application of the knowledge about such stress-responsive WOX genes has great potential to the genetic enhancement of stress tolerance in poplar and other commercially valuable woody perennial species

## 5 Conclusion

This research has introduced the first genome-wide examination of WOX transcription factor family in the desert poplar, *P. euphratica*, which is an adapted plant to stress. We discovered 18 genes in the *PeuWOX* family and determined their phylogenetic conservation, promoter structure, and dynamic expression patterns during drought and salt stress. We have found that the *PeuWOX* genes are at the center of an extensive developmental reprogramming network upon which the abiotic stress resilience of this species is built. The main stress- and hormone-sensitive cis-elements that are overrepresented in the promoters of *PeuWOX* offer a direct molecular connection between the environmental cues and changes in development. The expression pattern levels also revealed that there are different *PeuWOX* orchestrators of tissue-specific adaptative measures. Essentially, it has been shown that the fast and intense induction of *PeuWOX5* and *PeuWOX10* in the roots implies that the process plays a central role in the initiation of adventitious root growth to increase water and nutrient uptake in stressed conditions. The long-term upregulation of *PeuWOX4* and *PeuWOX17* observed in stems suggests a role in vascular cambium activity and secondary growth that is important in structural stability and water conductivity during long-term drought. An apparent transcriptional change was seen in the leaves; developmental genes (*PeuWOX1*, *PeuWOX2*, *PeuWOX4*, and *PeuWOX5*) rapidly declined to bring expansive growth to a halt, whereas stress-related genes such as *PeuWOX10* were increasingly expressed, which could be to activate cellular protection and dedifferentiation networks. These results would together suggest a model in which the *PeuWOX* family is involved in a very important tradeoff: under unpleasant conditions during abiotic stress, it is the developmental programs in aerial tissues that are suppressed, whereas the architecture of the root system and vascular development is stabilized by a set of concerted interactions between certain WOX members. This transcriptional re-prioritization seems to be among the root causes of the drought and salt tolerance of *P. euphratica*. Our study provides a resource base of future functional research such as genetic transformation and CRISPR-Cas9 genome editing, with *PeuWOX4*, *PeuWOX5*, *PeuWOX10*, and *PeuWOX17* identified as the best prospective genetic enhancement agents of stress tolerance in poplar and other woody perennials of economic importance.

## Data Availability

The desert poplar species data contributions obtainable in this study are freely available here: https://ngdc.cncb.ac.cn, GWHAAYU00000000.1.
